# A self-report measure of engagement with digital behavior change interventions (DBCIs): development and psychometric evaluation of the “DBCI Engagement Scale”

**DOI:** 10.1093/tbm/ibz039

**Published:** 2019-03-30

**Authors:** Olga Perski, Ann Blandford, Claire Garnett, David Crane, Robert West, Susan Michie

**Affiliations:** 1 Department of Clinical, Educational and Health Psychology, University College London, London, UK; 2 UCL Interaction Centre, University College London, London, UK; 3 Department of Behavioural Science and Health, University College London, London, UK

**Keywords:** Engagement, Digital behavior change interventions, Self-report scale, mHealth, eHealth, Psychometric evaluation, Alcohol reduction

## Abstract

Engagement with digital behavior change interventions (DBCIs) is a potentially important mediator of effectiveness; however, we lack validated measures of engagement. This study describes (a) the development of a self-report scale that captures the purported behavioral and experiential facets of engagement and (b) the evaluation of its validity in a real-world setting. A deductive approach to item generation was taken. The sample consisted of adults in the UK who drink excessively, downloaded the freely available *Drink Less* app with the intention to reduce alcohol consumption, and completed the scale immediately after their first login. Five types of validity (i.e., construct, criterion, predictive, incremental, divergent) were examined using exploratory factor analysis, correlational analyses, and through regressing the number of subsequent logins in the next 14 days onto total scale scores. Cronbach’s α was calculated to assess internal reliability. A 10-item scale assessing amount and depth of use, interest, enjoyment, and attention was generated. Of 5,460 eligible users, only 203 (3.7%) users completed the scale. Seven items were retained, and the scale was found to be unifactorial and internally reliable (α = 0.77). Divergent and criterion validity were not established. Total scale scores were not significantly associated with the number of subsequent logins (*B* = 0.02; 95% CI = −0.01 to 0.05; *p* = .14). Behavioral and experiential indicators of engagement with DBCIs may constitute a single dimension, but low response rates to engagement surveys embedded in DBCIs may make their use impracticable in real-world settings.

## Teaser

A new self-report scale that captures both the behavioural and experiential dimensions of digital engagement demonstrated promising psychometric properties in an initial evaluation study.

**Implications**

**Practice:** When deciding what measures of engagement to include in evaluations of the effectiveness of digital behavior change interventions (DBCIs), good psychometric properties must be carefully weighed against acceptability and measurement burden.

**Policy:** Resources should be directed toward further development and evaluation of engagement measures to arrive at validated instruments that facilitate easy comparison across DBCIs.

**Research:** Empirical tests of how behavioral and experiential facets of DBCI engagement relate to one another, both initially and over a period of time, will inform the development of validated measures of engagement.

## INTRODUCTION

Some degree of “engagement” with digital behavior change interventions (DBCIs) is logically necessary for them to be effective [1]. However, the association between the degree of engagement with such interventions and effectiveness is likely to depend on the intervention, the behavior, and the context in which the DBCI is used [2]. In practice, observed levels of engagement with DBCIs are typically considered too limited to support behavior change [3]. It has been estimated that less than 10% of health and fitness app users return to their selected app 7 days after registration [4,5], and a systematic review of health-related web-based intervention trials found that only 50% of participants engaged with interventions in the desired manner (i.e., interacting with all available intervention modules over a prespecified period of time), with estimates varying between 10% and 90% across trials [6]. Several studies, conducted across diverse behavioral domains and study settings, report a positive association of engagement with successful behavior change [7,8], suggesting that these variables may be linked through a dose-response function [1,9]. However, it is also plausible that those individuals who are more successful in achieving behavior change engage more, or that those who engage more differ systematically from those who are less engaged [2]. Despite various attempts to characterize the function relating engagement with successful behavior change [1,2,9,10], data cannot be aggregated efficiently due to the use of different definitions and measures of engagement (see [2] for a review of definitions). Hence, a psychometrically valid measure of engagement that facilitates easy comparison across DBCIs is needed. This study aimed to systematically develop such a measure and evaluate its validity it in a real-world setting.

As the design and evaluation of DBCIs requires knowledge of intervention content, computer programming, and design principles, the question as to what it means for someone to be engaged with a DBCI has been of great interest to psychologists, behavioral scientists, and interaction designers alike. Broadly, interaction designers have focused on the subjective experience of “flow” or “immersion” that occurs during the human–computer interaction, characterized by focused attention, intrinsic interest, balance between challenge and skill, transportation to a “different place” (e.g., the game environment), and loss of time and self-consciousness [11,12]. Psychologists have traditionally defined engagement as technology usage, perceived as a proxy for participant exposure to a DBCI’s “active ingredients” or component behavior change techniques [13,14]. More recently, however, the user’s subjective experience during DBCI usage has been acknowledged as a key dimension of engagement [1,15]. An interdisciplinary, integrative review thus suggests that engagement can be defined as (a) the extent of DBCI use (e.g., amount and depth of use) and (b) a subjective experience with emotional and cognitive facets (i.e., attention, interest, and affect) [2]. Engagement with a DBCI is hence thought to be conceptually distinct not only from “flow” and “immersion,” but also from pure technology usage.

Although many measures of engagement are currently in use (see [1,2] for overviews), including self-report scales and objective usage data, an instrument that captures both the behavioral and experiential facets of engagement is lacking. For example, although the *User Engagement Scale* [16], the *eHealth Engagement Scale* [17], the *Flow State Scale* [18], the *Immersion Experience Questionnaire* [19], the *Personal Involvement Inventory* [20], and the *Mobile Application Rating Scale* [21] capture a range of experiential facets (e.g., stimulation, enjoyment), they do not consider the behavioral facets of engagement (see Electronic Supplementary Material 1 for an overview of extant self-report scales). Automatically recorded usage data have typically been employed as a behavioral index of engagement [22–25], but it is unclear whether such records provide a valid measure of the experiential facets of engagement (e.g., attention). A validated measure of engagement that could be used by researchers, health care practitioners, and industry professionals, irrespective of them having access to the DBCI’s raw data, would be practically useful. Therefore, the present study aimed to develop and validate a new self-report scale that captures both the behavioral and experiential facets of engagement.

## MATERIALS AND METHODS

### Scale development

A detailed description of how the construct of interest was developed can be found in Electronic Supplementary Material 2.

### Item generation

A deductive approach to item generation was taken, meaning that the theoretical definition of the construct is used as a guide to generate scale items [26]. An initial pool of 18 items was generated by the first author based on the theoretical definitions of the five purported indicators of engagement (i.e., “amount of use,” “depth of use,” “attention,” “interest,” “enjoyment”). To mimic everyday language, items were designed to capture the intensity of the relevant thoughts, feelings, and behaviors (e.g., “How strongly did you experience enjoyment?”; “How much time do you roughly think that you spent on the app?”). Agreement on the set of initial items was reached through discussion between the co-authors. Although some of the items resemble those from existing scales (reviewed in Electronic Supplementary File 1), we did not explicitly draw on these. Our focus was on the development of items that demonstrate theoretical coherence, as opposed to novelty. Two items representing the authors’ “best bets” for a short measure of engagement were also developed (i.e., “How engaging was the app?”; “How much did you like the app?”).

### Item scaling

As the questionnaire was designed to be administered online and accessed through platforms with potentially small screens (e.g., smartphones), seven-point scaling was used where possible, with higher scores indicating greater intensity of engagement. Scale end- and mid-points were anchored to contextualize the response options: “not at all”; “moderately”; “extremely” [27].

### Content validity

Following the methodology in [28] and [29], a group of 10 behavioral scientists and 10 human–computer interaction experts were recruited from the authors’ networks (i.e., “experts”), and a group of 50 adult respondents recruited through Amazon’s Mechanical Turk (i.e., “non-experts”) were invited to complete a “content adequacy task” to determine the scale’s content validity. Respondents were asked to classify the randomly ordered items into one of six categories (i.e., “amount of use,” “depth of use,” “interest,” “attention,” “enjoyment,” plus an “unclassified” category). The task was hosted on Qualtrics [30] and was completed remotely without any researcher input. A minimum of 70% of respondents had to correctly classify an item for it to be retained [28,29].

Of the 18 initial items, two items tapping “interest,” three items tapping “attention,” five items tapping “enjoyment,” and one item tapping “amount of use” were correctly classified by a minimum of 70% of respondents in both groups (see Electronic Supplementary Material 3). To achieve balance across the five indicators, only the three highest performing items tapping “enjoyment” were retained. One item tapping “depth of use” was retained despite not reaching the *a priori* threshold of 70%; as “depth of use” is considered a necessary condition for engagement and one item tapping this facet was correctly classified by 65% of experts and 66% of non-experts, it was considered important to retain this item. In total, ten items were retained to form the *DBCI Engagement Scale* (see Electronic Supplementary Material 4).

### Scale evaluation

A preregistered protocol can be found on the Open Science Framework (OSF; see http://osf.io/qcmx4). Ethical approval was granted by UCL’s Departmental Research Ethics Committee (UCLIC/1213/015).

### Inclusion criteria

Participants were eligible to take part in the validation study if they had: (a) downloaded the alcohol reduction app *Drink Less* (see [31] for a detailed description of the app’s content) onto an iPhone or iPad during the study period (May 17, 2017 to March 6, 2018); (b) not opted out from allowing their data to be used for research purposes; (c) reported being 18 years of age or older; (d) reported residing in the United Kingdom (UK); (e) confirmed that they intended to reduce their drinking through responding “Interested in drinking less alcohol” to the question: “Why are you using *Drink Less*?”; and (f) reported an Alcohol Use Disorders Identification Test (AUDIT) score of 8 or more (indicating excessive alcohol consumption [32]). Eligibility was determined during app registration. The *Drink Less* app was selected because it includes evidence-based behavior change techniques, it has been designed with user-input, it is freely available on the UK Apple App Store, and the authors have access to the app’s usage data.

### Sampling

As app users are most likely to disengage after their first login session [4,5], novice users who had just downloaded the *Drink Less* app were recruited. The study was not publicly advertised. Interested participants identified the app on the Apple App Store or through word-of-mouth.

### Sample size

Due to the scarcity of prior research, it was not possible to predict what parameter estimates to expect. We therefore aimed to recruit a minimum of 200 participants, as this has been recommended as a rule-of-thumb for confirmatory factor analysis (CFA) [26].

### Measures

In addition to the *DBCI Engagement Scale*, data were collected on: (a) gender; (b) type of work (i.e., manual, non-manual, other); and (c) location during first use of the *Drink Less* app (i.e., home, work, vehicle, public transport, restaurant/pub/café, other’s home, can’t remember, other).

To allow the assessment of the scale’s criterion, predictive, and incremental validity, app screen views were automatically recorded, stored in an online database (NodeChef), and extracted using the free python library *pandas* to calculate objective “amount of use,” “depth of use,” and “number of subsequent logins.” The variable “amount of use” was derived by calculating the time spent (in seconds) during participants’ first login session. The variable “depth of use” was operationalized as the number of app modules visited during participants’ first login session, indexed as a proportion of the number of available modules (i.e., Goal Setting; Self-monitoring/Feedback; Action Planning; Normative Feedback; Cognitive Bias Re-Training; Identity Change; Other [31]). A new login was defined as a new screen view after 30 minutes of inactivity [33]. Participants were also asked to respond to the two “best bets” for a short measure of engagement (described above).

To allow the assessment of the scale’s divergent validity, participants were asked to respond to two items tapping the state of “flow” [11], as this was conceptualized as a qualitatively distinct state. Although engagement with DBCIs is expected to share some experiential qualities with the state of flow (i.e., “attention,” “interest”), users will not necessarily experience “balance between challenge and skill” or “loss of time and self-consciousness” when engaging with a DBCI. Therefore, assessing whether users can experience engagement without necessarily experiencing the state of flow was considered a useful test of the scale’s divergent validity. Two items from the *Flow State Scale* [18], measured on five-point Likert scales, that had previously been found to load most strongly onto the general flow factor were therefore selected (i.e., “When using *Drink Less*, the way time passed seemed to be different from normal”; “When using *Drink Less*, I was not worried about what others may have been thinking of me”). Although the original *Flow State Scale* is made up of 36 items, we only included two of its most strongly loading items to minimize measurement burden.

### Procedure

Eligible participants were prompted to fill out the *DBCI Engagement Scale* immediately after their first login session. Use of the smartphone’s home button to exit *Drink Less* triggered a local push notification with a link to the scale. Participants were asked to read the information sheet and provide informed consent prior to completing the scale. The push notification contained the following message: “Help science by responding to a brief survey.” Due to slow recruitment (i.e., ~3 responses/week), the message was changed on August 9, 2017 to: “Take a brief survey and enter a prize draw to win one of thirty £10 Amazon vouchers.” This incentive was chosen as the literature suggests that participants in online surveys respond at least as well to prize draws as other incentives [34]. This resulted in an average response rate of 5.5 responses/week, although it should be noted that this time period included the New Year period, in which there was an isolated spike of responses.

### Data analysis

All analyses were conducted using SPSS version 20.0 [35]. The assumptions for parametric tests were assessed (e.g., normality of the distribution of residuals). When these assumptions were violated, normalization was used (e.g., *z*-normalization for positively skewed data). Descriptive statistics (e.g., mean, range, variance) were calculated for each of the scale items and the additional variables of interest to determine suitability for factor analysis.

### Construct validity

It was hypothesized that a five-factor solution (i.e., “amount of use,” “depth of use,” “interest,” “attention,” “enjoyment”) would provide the best fit of the observed data. Preplanned analyses registered on the OSF therefore included the use of CFA. However, due to potential range restriction in key outcome variables resulting from self-selection during the recruitment process (i.e., only a small number of eligible users completing the scale), exploratory factor analysis (EFA) was deemed more suitable. The inspection of scree plots and eigenvalues >1 were used to determine the number of factors to retain [36]. Preplanned analyses also included a comparison of the fit of the CFA solution using the self-reported data as input with a CFA solution using a combination of self-reported data (i.e., the experiential indicators) and automatically recorded usage data (i.e., the behavioral indicators). However, an additional EFA was deemed more suitable.

### Internal consistency reliability

Cronbach’s α was calculated to assess internal consistency reliability. A large coefficient (i.e., .70 or above) was interpreted to indicate that there is strong item covariance [29].

### Criterion validity

Criterion validity was assessed by calculating Pearson’s correlation coefficient for the relationship between participants’ automatically recorded usage data from their first login (i.e., objective “amount of use” and “depth of use”) with their self-reported “amount of use” and “depth of use”, and with their total scale scores.

### Predictive validity

Preplanned analyses registered on the OSF included a regression analysis in which the outcome variable “subsequent login” (i.e., whether or not participants ever logged in again) would be regressed onto total scale scores. As all but 3.4% (7/203) of participants logged in again after their first session, this variable would have failed to discriminate between participants. We therefore conducted an unplanned analysis in which the variable “number of subsequent logins,” operationalized as the total number of logins in the 14 days after app registration, was regressed onto total scale scores. A cutoff at 14 days post-registration was deemed appropriate as DBCI access tends to be most prevalent during this time window [37].

### Incremental validity

Incremental validity was assessed through examining the additional variance accounted for in “number of subsequent logins” after adding the self-reported experiential indicators (but not the self-reported behavioral indicators) to a model including only the automatically recorded behavioral indicators of engagement.

### Divergent validity

The two items tapping “flow” were used to assess the scale’s divergent validity. Each item was correlated with participants’ total scale scores.

### Sensitivity analyses

As only a small proportion of eligible participants completed the scale, an unplanned sensitivity analysis was required to examine whether there was potential range restriction in the scale items and key outcome variables. A Mann–Whitney *U-*test was conducted to assess whether the median number of subsequent logins differed between those who did and did not complete the scale. An additional unplanned sensitivity analysis was conducted to assess if participants’ AUDIT scores were significantly associated with total scale scores or the number of subsequent logins.

## RESULTS

### Participant characteristics

During the study period (294 days; May 17, 2017 to March 6, 2018), a total of 8,336 users downloaded the *Drink Less* app, of which 5,460 (65.5%) were eligible to complete the scale. Of these, 311 (5.7%) users initiated the scale (i.e., opened the push notification), with 203 (3.7%) users completing it (see Electronic Supplementary Material 5). Participant demographic and drinking characteristics are reported in Table 1.

**Table 1:** | Participant demographic and drinking characteristics

Demographic characteristics	Completed scale (*N* = 203)	Initiated (but not completed) scale (*N* = 108)	*p* ^a^
Female, % (*N*)	64% (129)	53% (57)	.07
Type of work, % (*N*)			.85
Non-manual, % (*N*)	75% (152)	73% (79)	
Manual, % (*N*)	11% (22)	11% (11)	
Other, % (*N*)	14% (29)	17% (18)	
Age in years, mean (*SD*)	41.8 (10.7)	42.4 (9.5)	.66
Drinking characteristics			
AUDIT score, mean (*SD*)	17.6 (6.1)	18.3 (6.8)	.31

*AUDIT* Alcohol Use Disorders Identification Test

^a^Differences between groups were assessed using Chi-square tests or *t*-tests, as appropriate.

### Descriptive statistics for scale items

Descriptive statistics for the scale items are reported in Table 2. The majority of participants completed the scale at home (83%) or at work (7.9%). To account for observed skewness, *z*-score transformation was applied to the ten scale items, the two items used to test the scale’s criterion validity, and the three items used to test the scale’s predictive and incremental validity. Interitem correlations of the normalized items are reported in Table 3.

**Table 2:** | Descriptive statistics for the scale items (*N* = 203)

	Range	Mean (*SD*)	Variance	Skewness	Kurtosis
Scale items					
1. “How strongly did you experience interest?”	1–7	5.43 (1.19)	1.41	−0.61	0.34
2. “How strongly did you experience intrigue?”	1–7	5.05 (1.57)	2.48	−0.70	−0.27
3. “How strongly did you experience focus?”	2–7	5.06 (1.24)	1.54	−0.52	−0.07
4. “How strongly did you experience inattention?” (R)	1–7	5.32 (1.47)	2.17	−0.92	0.22
5. “How strongly did you experience distraction?” (R)	1–7	5.30 (1.65)	2.72	−0.91	−0.13
6. “How strongly did you experience enjoyment?”	1–7	4.30 (1.40)	1.95	−0.31	−0.37
7. “How strongly did you experience pleasure?”	1–7	3.63 (1.56)	2.44	0.07	−0.85
8. “How strongly did you experience annoyance?” (R)	1–7	5.77 (1.40)	1.97	−1.27	1.10
9. “Which of the app’s components did you visit?”	14.29–100.00	53.34 (22.99)	528.97	0.20	−0.73
10. “How much time do you roughly think that you spent on the app?” (seconds)	120–3,600	561.87 (379.07)	143,697.47	3.62	22.65
Items used to test criterion validity					
11. Objective depth of use	14.29–100.0	77.62 (16.69)	278.68	−0.66	0.40
12. Objective amount of use (seconds)	0–3,303	802.57 (646.03)	417,354.87	1.96	3.98
Items used to test predictive/incremental validity					
13. Number of subsequent logins	0–67	15.40 (12.35)	152.51	1.39	2.66
14. “How engaging was the app?”	1–7	5.15 (1.16)	1.34	−0.83	1.39
15. “How much did you like the app?”	2–7	5.33 (1.11)	1.23	−0.50	−0.14
Items used to test divergent validity					
16. “When using Drink Less, the way time passed seemed different from normal.”	1–5	2.87 (0.73)	0.53	−0.56	0.93
17. “When using Drink Less, I was not worried about what others may have been thinking about me.” (R)	1–5	2.78 (1.21)	1.47	0.10	−1.05

The symbol (R) indicates that values have been reverse scored prior to the calculation of descriptive statistics.

**Table 3:** | Interitem correlation matrix (*N* = 203)

Scale items	1.	2.	3.	4.	5.	6.	7.	8.	9.	10.
1. Interest	1									
2. Intrigue	.56**	1								
3. Focus	.73**	.53**	1							
4. Inattention (R)	.11	−.00	.14	1						
5. Distraction (R)	.10	−.02	.14*	.60**	1					
6. Enjoyment	.43**	.57**	.40**	−.08	−.15*	1				
7. Pleasure	.29**	.41**	.31**	−.25**	−.30**	.62**	1			
8. Annoyance (R)	.24**	.14*	.27**	.43**	.44**	.09	.02	1		
9. Which of app’s components	.17*	.27**	.13	.03	.04	.20**	.14*	.11	1	
10. How much time spent	.19**	.16*	.13	−.23**	−.17*	.16*	.19**	−.06	.28**	1

The symbol (R) indicates that values have been reverse scored prior to analysis.

*p < .05; **p < .001

### Construct validity

The Keiser–Meier Olkin (KMO) Test of Sampling Adequacy (KMO = 0.76) and Bartlett’s Test of Sphericity (*p* < .001) indicated that data were suited for factor analysis [38].Solution 1An EFA with oblique rotation indicated that a two-factor solution, accounting for 54.1% of the variance, provided the best fit of the observed data. However, the second factor was comprised only of the negatively worded items (4, 5, and 8), making little theoretical sense. On the basis of the conceptual parsimony of a one-factor solution, the second factor and the negatively worded items were discarded. Solution 2A subsequent EFA with oblique rotation indicated that a one-factor solution accounted for 44.5% of the variance (see Table 4).

**Table 4:** | Factor structure matrix for the DBCI Engagement Scale

Scale items	Solution 1^a^		Solution 2^b^	Solution 3^c^	
	Factor 1	Factor 2	Factor 1	Factor 1	Factor 2
1. Interest	**0.51**	0.14	**0.53**	**0.74**	0.05
2. Intrigue	**0.65**	0.02	**0.67**	**0.76**	0.07
3. Focus	**0.49**	0.20	**0.50**	**0.72**	0.01
4. Inattention (R)	−0.15	**0.79**	N/A	N/A	N/A
5. Distraction (R)	−0.21	**0.78**	N/A	N/A	N/A
6. Enjoyment	**0.86**	−0.09	**0.85**	**0.71**	−0.03
7. Pleasure	**0.60**	−0.32	**0.70**	**0.56**	−0.09
8. Annoyance (R)	0.11	**0.56**	N/A	N/A	N/A
9. Which of app’s components	**0.26**	0.03	**0.28**	N/A	N/A
10. How much time spent	**0.25**	−0.23	**0.26**	N/A	N/A
11. Objective depth of use	N/A	N/A	N/A	0.10	**0.73**
12. Objective amount of use	N/A	N/A	N/A	−0.09	**0.64**

The symbol (R) indicates that values have been reverse scored prior to analysis. Factor loadings of 0.25 and greater are in bold.

^a^EFA with oblique rotation, including items 1–10.

^b^EFA with oblique rotation, including items 1, 2, 3, 6, 7, 9, and 10.

^c^EFA with oblique rotation, including items 1, 2, 3, 6, 7, 11, and 12

Solution 3An EFA with oblique rotation using a combination of self-reported data (i.e., items 1, 2, 3, 6, and 7) and automatically recorded data (i.e., items 11 and 12) suggested a two-factor solution, which accounted for 63.5% of the variance. The experiential indicators loaded clearly onto factor 1, and the behavioral indicators loaded clearly onto factor 2 (see Table 4).

Solution 2 was selected for further analysis, as it contained only the self-reported items. Prior to further analyses, a total scale score was calculated for each participant, with equal weight given to each of the retained items (i.e., 1, 2, 3, 6, 7, 9, 10).

### Internal consistency reliability

Internal consistency estimates for the seven-item scale yielded a coefficient α of 0.77, indicating adequate internal consistency reliability [29].

### Criterion validity

Total scale scores were significantly correlated with objectively recorded “depth of use,” *r*(201) = .23, *p* < .01, but not with objectively recorded “amount of use,” *r*(201) = −.02, *p* = .82. Self-reported “depth of use” was significantly correlated with objectively recorded “depth of use,” *r*(201) = .44, *p* < .001. Self-reported “amount of use” was significantly correlated with objectively recorded “amount of use,” *r*(201) = .15, *p* < .05.

### Predictive validity

As shown in Table 5, total scale scores did not significantly predict the number of subsequent logins (*B* = 0.02; 95% CI = −0.01, 0.05; *p* = .14). Asking users about how engaging they thought the app was (*B* = 0.07; 95% CI = −0.07, 0.21; *p* = .30) or how much they liked the app (*B* = 0.09; 95% CI = −0.05, 0.22) did not significantly predict the number of subsequent logins. A post hoc power analysis indicated that a total of 203 participants provided 44% power (two-tailed α = .05) to detect a regression coefficient of 0.02 for the association between total scale scores and the number of subsequent logins [39].

**Table 5:** | Univariable and multivariable linear regression models predicting the number of subsequent logins

	Beta (95% CI)	*p*
Predictive validity		
Total scale scores	0.02 (−0.01, 0.05)	.14
How engaging was the app?	0.07 (−0.07, 0.21)	.30
How much did you like the app?	0.09 (−0.05, 0.22)	.20
Incremental validity		
Model 1		
Objective amount of use	0.07 (−0.09, 0.22)	.40
Objective depth of use	0.03 (−0.13, 0.18)	.75
Model 2		
Objective amount of use	0.09 (−0.07, 0.25)	.27
Objective depth of use	−0.01 (−0.17, 0.15)	.89
Interest	0.25 (0.03, 0.46)	.02*
Focus	−0.10 (−0.30, 0.11)	.35
Enjoyment	0.02 (−0.18, 0.22)	.86
Intrigue	0.04 (−0.15, 0.22)	.71
Pleasure	−0.02 (-0.20, 0.15)	.79

*p < .05

### Incremental validity

A model including the automatically recorded indicators of engagement (i.e., items 11 and 12) accounted for 0.7% of variance in the number of subsequent logins. Neither objective “amount of use” nor objective “depth of use” were significant predictors of the number of subsequent logins (see Table 5). A model including the automatically recorded behavioral indicators in addition to the experiential indicators of engagement (i.e., items 1, 2, 3, 6, 7) accounted for 4.9% of variance in the number of subsequent logins. Interest was the only significant predictor of the number of subsequent logins.

### Divergent validity

Total scale scores were significantly correlated with the first (“When using *Drink Less*, the way time passed seemed different from normal”) but not the second (“When using *Drink Less*, I was not worried about what others may have been thinking about me”) item tapping “flow” (*r*(201) = .14; *p* = .04 and *r*(201) = −.07; *p* = .33, respectively). The two items tapping flow were not significantly correlated with one another in this sample (*r*(201) = −.02; *p* = .82).

### Sensitivity analysis

The first sensitivity analysis indicated that those who completed the scale had a significantly greater median number of subsequent logins (median = 13.0, interquartile range (IQR) = 6.0–21.0) than eligible users who did not complete the scale (median = 6.0, IQR = 1.0–16.0), *U* = 361,135.5, *p* < .001. The second sensitivity analysis showed that participants’ AUDIT scores were neither significantly correlated with total scale scores (*r*(201) = .10; *p* = .14) nor with the number of subsequent logins (*r*(201) = .004; *p* = .95).

## DISCUSSION

This study described the systematic development of a new self-report measure of engagement with DBCIs and its validation in a real-world setting with an alcohol reduction app. As fewer than 5% of eligible users completed the scale, our first observation is that we have not established that it is feasible to measure engagement through self-report in a real-world setting. Second, results from a series of EFAs indicate that the seven-item *DBCI Engagement Scale* is unifactorial and internally reliable. Third, total scale scores were significantly but weakly correlated with objective “depth of use” but not significantly correlated with objective “amount of use”, thus questioning the scale’s criterion validity. Fourth, total scale scores did not predict the number of subsequent logins in the next 14 days. Finally, total scale scores were significantly associated with one of the two items from the *Flow State Scale*, which questions the scale’s divergent validity.

These results should be interpreted in the light of a number of important methodological and theoretical limitations. Through comparing the number of subsequent logins between the analytic sample and the sample of total eligible users, it was evident that the analytic sample was biased toward highly engaged users. It is likely that this restricted the range in both scale items and key outcome variables, thus limiting the ability of the present study to evaluate the scale’s validity. Our inclusion criteria (i.e., expressing a desire to reduce drinking, being willing to use an app, being willing to share data with the researchers) may also have contributed to the apparent self-selection bias. However, these inclusion criteria mirror those in randomized controlled trials of health apps [31,40]. It is notoriously difficult to study engagement in real-world settings, as highly engaged individuals are more likely to take part in such research (i.e., users who login more frequently have a greater likelihood of responding to follow-up surveys) [41]. An important avenue for future research is therefore to evaluate the scale’s validity in a more controlled setting, with a view to recruiting participants with a broader range of engagement levels (e.g., students taking part in research for credit). The authors are currently in the process of evaluating the scale in an online sample taking part in the research in exchange for a financial reward.

The observation that the negatively worded items (e.g., “inattention,” “distraction”) were found to load onto a second factor in the initial EFA (which resulted in the removal of these items) suggests that participants may have found it difficult to respond to the negatively worded items. Despite having assessed the items’ content validity through an initial content adequacy task, it is possible that “inattention” is not seen as the polar opposite of “attention” in everyday language. Future work using cognitive interviewing techniques is therefore required to further refine the scale items, ensuring that the retained items are easy to respond to [42]. Moreover, the observation that the two items assessing the state of flow were not significantly correlated in this sample also highlights the importance of using well-validated scales when benchmarking a new scale, where available.

The lack of a significant association of initial experiential and behavioral engagement with future engagement can be interpreted in multiple ways. First, the study was not adequately powered to detect a weak relationship between initial and future engagement. Second, it is plausible that other factors, such as motivation to change or perceived personal relevance, are in fact more strongly predictive of future engagement than initial experiential and behavioral engagement. Indeed, systematic reviews of DBCIs indicate that aggregate measures of engagement (e.g., total number of logins over a period of time) are influenced by attributes of the DBCI itself (e.g., tailoring, aesthetics), characteristics of the users (e.g., motivation to change), and the context in which the DBCI is used (e.g., social cues) [2,43]. Future attempts to validate state-based measures of experiential and behavioral engagement should also carefully consider other indicators of predictive validity. For example, it is plausible that greater intensity of initial engagement predicts knowledge or skills at a future time point, as suggested by the Elaboration Likelihood Model of Persuasion [44].

Third, it is also plausible that our construct definition did not adequately capture “engagement.” For example, it has been argued that engagement with DBCIs extends beyond technology-based experiential and behavioral indicators, as there may be periods during which an individual is still engaged in the behavior change process (e.g., mentally rehearsing the successful performance of the behavior) but is no longer using the technology to facilitate this [1]. Other theorists have argued that engagement includes additional cognitive facets (e.g., the ability to comprehend the intervention materials and retain key information) [45], or that it does not include any cognitive or emotional facets beyond technology usage [46]. As it is impossible to objectively determine the theoretical foundation of psychological constructs [47], the lack of consensus as to what engagement is, to be expected. Even without this consensus, empirical tests of how key variables relate to one another, both initially and over a period of time, are critically important in the process of developing an operational definition of engagement that is practically useful for researchers, practitioners, and policy makers.

In line with theories of behavior change [48], engagement with DBCIs may be more usefully conceptualized as a behavior that is influenced by multiple, dynamically interacting intra- and extra-individual factors (e.g., psychological, social, environmental). It may be more fruitful to consider how different configurations of intra- and extra-individual factors dynamically interact over short time periods (e.g., hours, days) to influence behavior (sometimes referred to as “state-space representations” of when a particular intervention produces a given effect) [49]. Within such a framework, the likelihood that a user engages behaviorally with an alcohol reduction app may increase if, for example, (a) their memory of the app being interesting/enjoyable is salient, (b) their daily level of motivation to reduce drinking is high, and (c) they are not surrounded by others who drink. The interrelationships between such variables should be tested using experience sampling methodology to gather temporally rich, contextualized data [50], which can be modeled using computational techniques from control systems engineering (e.g., dynamic systems modeling) [51].

In conclusion, behavioral and experiential indicators of engagement may resolve to a single dimension, but low response rates to engagement surveys embedded in DBCIs may make their use impracticable in real-world settings. The lack of an association between total scale scores and the number of subsequent logins suggests that other factors, such as motivation to change, may play a more important role in the prediction of future engagement than initial behavioral and experiential engagement.

## Supplementary Material

ibz039_suppl_Supplementary_Material-1

ibz039_suppl_Supplementary_Material-2

ibz039_suppl_Supplementary_Material-3

ibz039_suppl_Supplementary_Material-4

ibz039_suppl_Supplementary_Material-5
