# Supplementary material for: A self-report measure of engagement with digital behavior change interventions (DBCIs): development and psychometric evaluation of the “DBCI Engagement Scale”
Source: Transl Behav Med. 2019 Mar 30;10(1):267–77. doi: 10.1093/tbm/ibz039 (PMC8411853; doi:10.1093/tbm/ibz039)
Supplement: ibz039_suppl_Supplementary_Material-1 [file TBM_10_1_267_s1.docx]

**Electronic Supplementary Material 1**

Scoping review of psychometric properties of existing self-report measures of engagement with DBCIs.

| Self-report scale | Description | Construct validity | Reliability | Criterion validity | Divergent validity | Predictive validity |
| --- | --- | --- | --- | --- | --- | --- |
| User Engagement Scale [1] | A 123-item scale, designed to measure the following 10 sub-dimensions of engagement: ‘aesthetics’, ‘affect’, ‘focused attention’, ‘challenge’, ‘control’, ‘feedback’, ‘interest’, ‘motivation’, ‘novelty’ and ‘perceived time’. | Exploratory Factor Analysis (EFA) indicated a six-factor solution: ‘focused attention’, ‘perceived usability’, ‘aesthetics’, ‘endurability’, ‘novelty’, and ‘felt involvement’.  The factor solution did not replicate in a new sample [2]. The authors recognised that their definition of engagement contains attributes that predict, rather than are part of, the focal construct. | Cronbach’s α was calculated to assess internal consistency reliability for each factor, ranging from .72-.90. | Not assessed. | Not assessed. | Not assessed. |
| eHealth Engagement Scale [3] | A 12-item scale, designed to assess the following sub-dimensions of engagement with digital health information: ‘absorbing’, ‘attention-grabbing’, ‘stimulating’, ‘surprising’, ‘suspenseful’, ‘thought-provoking’, ‘clever’, ‘convincing’, ‘balanced’, ‘believable’, ‘dull’ and ‘hip/cool’. | Confirmatory Factor Analysis (CFA) indicated acceptable fit of a four-factor model: ‘involving’, ‘credible’, ‘dull’ and ‘hip/cool’. | Cronbach’s α was calculated to assess internal consistency reliability for each factor but is not reported. | Not assessed. | Not assessed. | Assessed the scale’s ability to predict aggregate scores on three proximal outcomes (e.g. “The information made me feel more confident that I can do something”). The four-factor solution accounted for 56% of variance in the proximal outcome. |
| Flow State Scale [4] | A 54-item scale, designed to measure the following 9 sub-dimensions of the state of ‘flow’ [5]: ‘challenge-skill’, ‘action-awareness’, ‘clear goals’, ‘unambiguous feedback’, ‘concentration’, ‘sense of control’, ‘loss of self-consciousness’, ‘transformation of time’ and ‘autoletic experience’. | A series of CFAs, resulting in the removal of 18 items, indicated that the a priori nine-factor structure was supported. | Cronbach’s α was calculated to assess the internal consistency reliability for each factor, ranging from .80-.86. | Not assessed. | Not assessed. | Not assessed. |
| Immersion Experience Questionnaire [6] | A 33-item scale, designed to measure 8 sub-dimensions of the state of ‘immersion’ during digital game-play: ‘temporal dissociation’, ‘focused immersion’, ‘heightened enjoyment’, ‘control and autonomy’, ‘curiosity’, ‘emotional involvement’, ‘transportation to a different place’ and ‘attention’. | EFA indicated a five-factor solution: ‘cognitive involvement’, ‘real world dissociation’, ‘challenge’, ‘emotional involvement’ and ‘control’. | Not assessed. | Not assessed. | Not assessed. | Not assessed. |
| Personal Involvement Inventory [7, 8] | 30-item scale, designed to measure the ‘motivational state of involvement’ with different commercial products [8], measured using bipolar adjectives (e.g. important-unimportant, boring-interesting). | EFA, after removing 10 items, indicated a one-factor solution. | Test-retest reliability indicated that item-to-item correlations between Time 1 and Time 2 (3 weeks later) ranged from .31-.93. | Scale scores for products (e.g. car, jeans) were found to correspond to previous classifications of such products into low or high involvement categories. | Not assessed. | Not assessed. |
| Mobile Application Rating Scale [9] | 23-item scale, designed to function as a quality assessment tool for mobile health apps, assessing the following 4 sub-dimensions: ‘engagement’, ‘functionality’, ‘aesthetics’ and ‘information quality’. | Not assessed. | Inter-rater reliability, calculated using the intra-class correlation coefficient, ranged from .5-.83. Internal consistency reliability, calculated using Cronbach’s α, ranged from .80-.93. | Not assessed. | Not assessed. | Not assessed. |

**References**

1. O’Brien, H. L., & Toms, E. G. (2010). The Development and Evaluation of a Survey to Measure User Engagement. *Journal of the American Society for Information Science & Technology*, *61*(1), 50–69. doi:10.1002/asi

2. O’Brien, H. L., & Toms, E. G. (2013). Examining the generalizability of the User Engagement Scale (UES) in exploratory search. *Information Processing and Management*, *49*(5), 1092–1107. doi:10.1016/j.ipm.2012.08.005

3. Lefebvre, R. C., Tada, Y., Hilfiker, S. W., & Baur, C. (2010). The Assessment of User Engagement with eHealth Content: The eHealth Engagement Scale. *Journal of Computer-Mediated Communication*, *15*, 666–681. doi:10.1111/j.1083-6101.2009.01514.x

4. Jackson, S. A., & Marsh, H. W. (1996). Development and validation of a scale to measure optimal experience: The Flow State Scale. *Journal of Sport & Exercise Psychology*, *18*, 17–35. doi:10.1080/15298860309027

5. Csikszentmihalyi, M. (1990). *Flow: The psychology of optimal performance*. New York: Cambridge University Press.

6. Jennett, C., Cox, A. L., Cairns, P., Dhoparee, S., Epps, A., Tijs, T., & Walton, A. (2008). Measuring and Defining the Experience of Immersion in Games. *International Journal of Human-Computer Studies*, *66*(9), 641–661.

7. Zaichkowsky, J. L. (1994). The Personal Involvement Inventory: Reduction, Revision, and Application to Advertising. *Journal of Advertising*, *23*(4), 59–70. doi:10.1080/00913367.1943.10673459

8. Zaichkowsky, J. L. (1985). Measuring the Involvement Construct. *Journal of Consumer Research*, *12*(3), 341–352.

9. Stoyanov, S. R., Hides, L., Kavanagh, D. J., Zelenko, O., Tjondronegoro, D., & Mani, M. (2015). Mobile App Rating Scale: A New Tool for Assessing the Quality of Health Mobile Apps. *JMIR mHealth and uHealth*, *3*(1), e27. doi:10.2196/mhealth.3422
