# Supplementary material for: A self-report measure of engagement with digital behavior change interventions (DBCIs): development and psychometric evaluation of the “DBCI Engagement Scale”
Source: Transl Behav Med. 2019 Mar 30;10(1):267–77. doi: 10.1093/tbm/ibz039 (PMC8411853; doi:10.1093/tbm/ibz039)
Supplement: ibz039_suppl_Supplementary_Material-2 [file TBM_10_1_267_s2.docx]

### Electronic Supplementary Material 2

### Construct development

The construct of interest was developed through three iterative steps [1]: i) defining the conceptual domain to which the construct belongs (e.g. thought, feeling, behaviour, outcome); ii) defining the entity to which the construct applies (e.g. person, task, process, relationship) and how stable it is expected to be over time, across situations and across cases; and iii) defining the set of fundamental attributes or characteristics that are necessary and sufficient for something to be an instance of the construct. Two data sources were drawn upon to generate a definition of the construct: a systematic review of the behavioural science, computer science and human-computer interaction literatures (detailed methodology reported in [2]) and an empirical think aloud and interview study with potential users of smartphone apps for smoking cessation and alcohol reduction (detailed methodology, but not data pertaining to the present study, reported in [3]).

#### 1. Conceptual domain

Existing definitions of engagement identified in the systematic review could broadly be categorised into one of two conceptual domains: ‘engagement as subjective experience’, incorporating emotional and cognitive facets, and ‘engagement as behaviour’ [2]. A similar distinction was made by participants in the think aloud and interview study, who described feelings of enjoyment, stimulation and focus when engaging with a DBCI. These participants also described engagement as frequent DBCI use over time, thus highlighting the behavioural facet of engagement. Therefore, it was hypothesised that engagement spans two conceptual domains: an experiential domain (with cognitive and emotional facets) and a behavioural domain.

#### 2. Type of entity

As engagement has been found to vary within users over time and across DBCIs, often as a function of person- or technology-specific attributes (e.g. motivation to change, self-efficacy, tailoring, aesthetics) [2–5], it was hypothesised that engagement can usefully be conceived of as a state rather than a trait [6]. The two data sources did not help clarifying whether the state of engagement is best conceived of as a task-specific construct (i.e. applicable only in situations where a DBCI is present) or whether it extends to other objects. For the purpose of the present study, it was hypothesised that the state of engagement is task-specific, as this implies that it is sufficient to consider situations in which the object of interest is a DBCI, rather than any object.

#### 3. Necessary and sufficient conditions

Two behavioural indicators and three experiential indicators were identified as particularly important for determining the intensity of the state of engagement: amount of use, depth of use, attention, interest and enjoyment. First, spending time on a DBCI (i.e. ‘amount of use’) and accessing at least one of its components (i.e. ‘depth of use’) were both considered necessary for engagement. Spending time on a DBCI, though not actively using it (e.g. posting in an online forum), was considered necessary as research shows that ‘lurking’ in online discussion forums (i.e. reading others’ comments without actively contributing) can help people achieve behaviour change [7]. As research shows that unique behaviour change techniques are independently associated with successful behaviour change [8–10], the range of components accessed was considered necessary to determine the intensity of DBCI engagement. The behavioural indicators were hypothesised to be jointly insufficient for someone to be engaged, as a user may scroll through information on an app without paying attention to its content. Therefore, three experiential indicators were also considered necessary for engagement: paying attention to the DBCI (‘attention’), feeling interested in it (‘interest’) and experiencing enjoyment whilst using it (‘enjoyment’). It is widely accepted that the process of selective attention helps allocating limited resources to specific stimuli, and that the function of interest is to direct attention towards important stimuli [11–14]. Although the two data sources also indicated that enjoyment is a key aspect of engagement [2], it is unclear whether this is a necessary condition for someone to be engaged, as it is possible to pay attention to an app and be interested in its content without experiencing enjoyment. However, it was hypothesised that the two behavioural and three experiential indicators were necessary and jointly sufficient for engagement.

**References**

1. MacKenzie, S., Podsakoff, P., & Podsakoff, N. (2011). Construct Measurement and Validation Procedures in MIS and Behavioral Research: Integrating New and Existing Techniques. *MIS Quarterly*, *35*(2), 293–334. doi:10.2307/23044045

2. Perski, O., Blandford, A., West, R., & Michie, S. (2017). Conceptualising engagement with digital behaviour change interventions: a systematic review using principles from critical interpretive synthesis. *Translational Behavioral Medicine*, *7*, 254–267. doi:10.1007/s13142-016-0453-1

3. Perski, O., Blandford, A., Ubhi, H. K., West, R., & Michie, S. (2017). Smokers’ and drinkers’ choice of smartphone applications and expectations of engagement: a think aloud and interview study. *BMC Medical Informatics and Decision Making*, *17*(25), 1–14. doi:10.1186/s12911-017-0422-8

4. Milward, J., Drummond, C., Fincham-Campbell, S., & Deluca, P. (2018). What makes online substance-use interventions engaging? A systematic review and narrative synthesis. *Digital Health*, *4*, 1–25. doi:10.1177/2055207617743354

5. Brouwer, W., Kroeze, W., Crutzen, R., de Nooijer, J., de Vries, N. K., Brug, J., & Oenema, A. (2011). Which intervention characteristics are related to more exposure to internet-delivered healthy lifestyle promotion interventions? A systematic review. *Journal of medical Internet research*, *13*(1), e2. doi:10.2196/jmir.1639

6. Chaplin, W. F., John, O. P., & Goldberg, L. R. (1988). Conceptions of States and Traits: Dimensional Attributes With Ideals as Prototypes. *Journal of Personality and Social Psychology*, *54*(4), 541–557. doi:10.1037/0022-3514.54.4.541

7. Graham, A. L., Stanton, C. A., Papandonatos, G. D., & Erar, B. (2015). Use of an online smoking cessation community promotes abstinence: Results of propensity score weighting. *Health Psychology*, *34*, 1286–1295. doi:10.1037/hea0000278

8. Michie, S., Wood, C. E., Johnston, M., Abraham, C., Francis, J. J., & Hardeman, W. (2015). Behaviour change techniques: The development and evaluation of a taxonomic method for reporting and describing behaviour change interventions (a suite of five studies involving consensus methods, randomised controlled trials and analysis of qualitative da. *Health Technology Assessment*, *19*(99), 1–187. doi:10.3310/hta19990

9. Michie, S., Abraham, C., Whittington, C., & Mcateer, J. (2009). Effective Techniques in Healthy Eating and Physical Activity Interventions: A Meta-Regression. *Health Psychology*, *28*(6), 690–701. doi:10.1037/a0016136

10. Michie, S., Whittington, C., Hamoudi, Z., Zarnani, F., Tober, G., & West, R. (2012). Identification of behaviour change techniques to reduce excessive alcohol consumption. *Addiction*, *107*(8), 1431–1440. doi:10.1111/j.1360-0443.2012.03845.x

11. Posner, M. I., & Petersen, S. E. (1990). The Attention System of the Human Brain. *Annual Review of Neuroscience*, *13*, 25–42. doi:10.1146/annurev.neuro.13.1.25

12. Yiend, J. (2010). The effects of emotion on attention: A review of attentional processing of emotional information. *Cognition and Emotion*, *24*(1), 3–47. doi:10.1080/02699930903205698

13. Tomkins, S. S. (2008). *Affect Imagery Consciousness: The Complete Edition* (Vol. I–II). New York: Springer Publishing Company. doi:10.1192/bjp.109.463.837

14. Silvia, P. J. (2008). Interest - The Curious Emotion. *Current Directions in Psychological Science*, *17*(1), 57–60. doi:10.1111/j.1467-8721.2008.00548.x
