# Supplementary material for: A self-report measure of engagement with digital behavior change interventions (DBCIs): development and psychometric evaluation of the “DBCI Engagement Scale”
Source: Transl Behav Med. 2019 Mar 30;10(1):267–77. doi: 10.1093/tbm/ibz039 (PMC8411853; doi:10.1093/tbm/ibz039)
Supplement: ibz039_suppl_Supplementary_Material-3 [file TBM_10_1_267_s3.docx]

**Electronic Supplementary Material 3**

Description of experts’ (*N* = 20) and non-experts’ (*N* = 50) classifications of the initial 18-item scale.

| **Item (Intended Category)** | **Group** | **Interest (%)** | **Attention (%)** | **Enjoyment (%)** | **Amount of use (%)** | **Depth of use (%)** | **Unclassified (%)** |
| --- | --- | --- | --- | --- | --- | --- | --- |
| 1. “How strongly did you experience interest?” (Interest) | Experts | **100%** | 0% | 0% | 0% | 0% | 0% |
|  | Non-experts | **84%** | 4% | 10% | 0% | 0% | 2% |
| 2. “How strongly did you experience frustration?” (Enjoyment) | Experts | 5% | 0% | **90%** | 0% | 0% | 5% |
|  | Non-experts | 2% | 4% | **78%** | 0% | 8% | 8% |
| 3. “How strongly did you experience focus?” (Attention) | Experts | 0% | **95%** | 0% | 0% | 5% | 0% |
|  | Non-experts | 6% | **78%** | 0% | 2% | 8% | 6% |
| 4. “How strongly did you experience boredom?” (Interest) | Experts | 55% | 10% | 35% | 0% | 0% | 0% |
|  | Non-experts | 52% | 4% | 38% | 2% | 0% | 4% |
| 5. “How strongly did you experience inattention?” (Attention) | Experts | 0% | **100%** | 0% | 0% | 0% | 0% |
|  | Non-experts | 0% | **94%** | 0% | 4% | 0% | 2% |
| 6. “How strongly did you experience absorption?” (Attention) | Experts | 20% | 30% | 5% | 0% | 45% | 0% |
|  | Non-experts | 18% | 16% | 2% | 12% | 46% | 6% |
| 7. “How strongly did you experience annoyance?” (Enjoyment) | Experts | 5% | 0% | **90%** | 0% | 0% | 5% |
|  | Non-experts | 6% | 0% | **80%** | 0% | 8% | 6% |
| 8. “How strongly did you experience fascination?” (Interest) | Experts | 80% | 0% | 15% | 0% | 5% | 0% |
|  | Non-experts | 40% | 12% | 32% | 2% | 6% | 8% |
| 9. “How strongly did you experience distraction?” (Attention) | Experts | 0% | **85%** | 5% | 0% | 10% | 0% |
|  | Non-experts | 14% | **80%** | 0% | 2% | 4% | 0% |
| 10. “How strongly did you experience enjoyment?” (Enjoyment) | Experts | 0% | 0% | **100%** | 0% | 0% | 0% |
|  | Non-experts | 8% | 0% | **84%** | 2% | 0% | 6% |
| 11. “How strongly did you experience intrigue?” (Interest) | Experts | **80%** | 10% | 0% | 0% | 5% | 5% |
|  | Non-experts | **74%** | 6% | 12% | 2% | 2% | 4% |
| 12. “How strongly did you experience mindfulness?” (Attention) | Experts | 0% | 55% | 0% | 0% | 25% | 20% |
|  | Non-experts | 8% | 56% | 4% | 2% | 22% | 8% |
| 13. “How strongly did you experience fun?” (Enjoyment) | Experts | 5% | 0% | **95%** | 0% | 0% | 0% |
|  | Non-experts | 0% | 4% | **86%** | 4% | 0% | 6% |
| 14. “How strongly did you experience pleasure?” (Enjoyment) | Experts | 0% | 0% | **100%** | 0% | 0% | 0% |
|  | Non-experts | 2% | 2% | **92%** | 0% | 2% | 2% |
| 15. “How strongly did you experience indifference?” (Interest) | Experts | 60% | 0% | 25% | 0% | 0% | 15% |
|  | Non-experts | 46% | 4% | 16% | 2% | 8% | 22% |
| 16. “How much time (in minutes) do you roughly think that you spent on the app?” (Amount of use) | Experts | 0% | 0% | 0% | **85%** | 5% | 10% |
|  | Non-experts | 0% | 2% | 2% | **80%** | 8% | 8% |
| 17. “Which of the app’s components did you visit (e.g. diary, goal setting, game)?” (Depth of use) | Experts | 0% | 0% | 0% | 20% | 65% | 15% |
|  | Non-experts | 4% | 4% | 0% | 8% | 66% | 18% |
| 18. “Which component was most memorable?” (Depth of use) | Experts | 15% | 30% | 0% | 0% | 15% | 40% |
|  | Non-experts | 16% | 18% | 32% | 2% | 12% | 20% |

* Percentages in bold indicate items that were correctly classified by a minimum of 70% of respondents in both groups.
