# Supplementary material for: A self-report measure of engagement with digital behavior change interventions (DBCIs): development and psychometric evaluation of the “DBCI Engagement Scale”
Source: Transl Behav Med. 2019 Mar 30;10(1):267–77. doi: 10.1093/tbm/ibz039 (PMC8411853; doi:10.1093/tbm/ibz039)
Supplement: ibz039_suppl_Supplementary_Material-4 [file TBM_10_1_267_s4.docx]

**Electronic Supplementary Material 4**

*The ‘DBCI Engagement Scale’*

Please answer the following questions with regards to your most recent use of the *Drink Less* app.

How strongly did you experience the following?

1. Interest

2. Intrigue

3. Focus

4. Inattention

5. Distraction

6. Enjoyment

7. Annoyance

8. Pleasure

*Measured on a 7-point scale with end-points and middle anchored: not at all; moderately; extremely*

9. How much time (in minutes) do you roughly think that you spent on the app?

*Enter free text*

10. Which of the app’s components do you remember visiting? (You can select multiple options)

a) Calendar (Self-monitoring/feedback)

b) Create and view goals (Goal setting)

c) What has and hasn’t worked (Self-monitoring/feedback)

d) Create and view action plans (Action planning)

e) Your hangover and you (Self-monitoring/feedback)

f) Review your drinking (Normative feedback)

g) Dashboard (Self-monitoring/feedback)

h) Game (Cognitive bias re-training)

i) Drink + me (Identity change)

j) Useful information (Other)

k) Other (Other)

l) Can’t remember (Other)

*Indexed as a proportion of available modules (e.g. 5/7 * 100 = 71.4).*
