# Supplementary figures and images for: A self-report measure of engagement with digital behavior change interventions (DBCIs): development and psychometric evaluation of the “DBCI Engagement Scale”
Source: Transl Behav Med. 2019 Mar 30;10(1):267–77. doi: 10.1093/tbm/ibz039 (PMC8411853; doi:10.1093/tbm/ibz039)

**Electronic Supplementary Material 5**

*
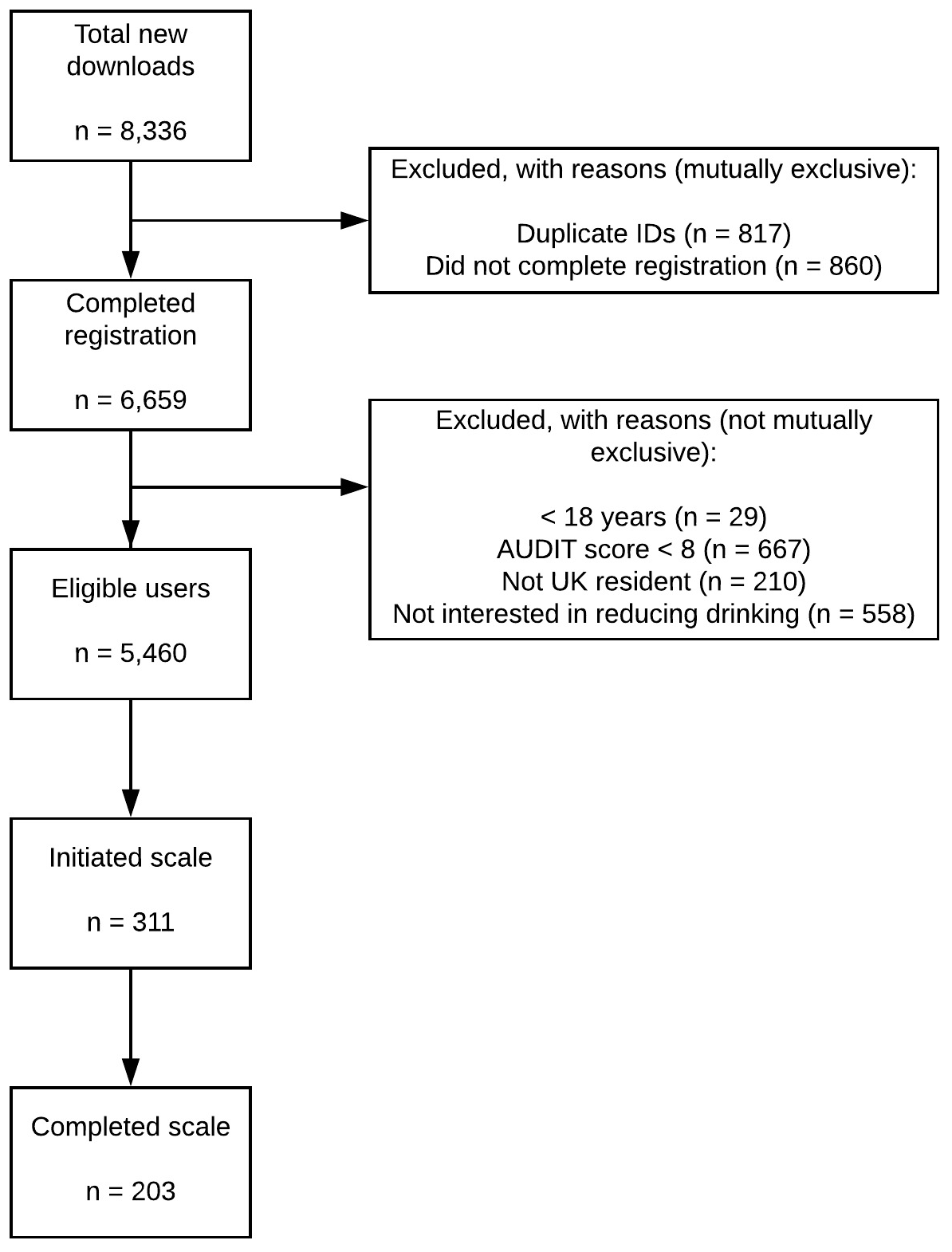
*

*Figure 1.* Participant flow chart.

Supplement: ibz039_suppl_Supplementary_Material-5 [file TBM_10_1_267_s5.docx]
